# Transcatheter Aortic Valve Implantation and Conduction Disturbances: Focus on Clinical Implications

**DOI:** 10.3390/jcdd10110469

**Published:** 2023-11-19

**Authors:** Antonios Halapas, Leonidas Koliastasis, Ioannis Doundoulakis, Christos-Konstantinos Antoniou, Christodoulos Stefanadis, Dimitrios Tsiachris

**Affiliations:** 1 Department of Interventional Cardiologist and THV Program, Athens Medical Center, 11526 Athens, Greece; ahalapas@gmail.com; 2 Department of Cardiology, Centre Hospitalier Universitaire Saint-Pierre, Université Libre de Bruxelles (ULB), 1000 Brussels, Belgium; lkoliastasis@gmail.com; 3 First Department of Cardiology, National and Kapodistrian University, “Hippokration” Hospital, 11527 Athens, Greece; ckantoniou@hotmail.gr (C.-K.A.); dtsiachris@yahoo.com (D.T.); 4 Athens Heart Centre, Athens Medical Centre, 11526 Athens, Greece; stefanadischristodoulos@gmail.com

**Keywords:** aortic stenosis, conduction abnormalities, pacemaker, transcatheter aortic valve implantation, TAVI

## Abstract

Transcatheter aortic valve implantation (TAVI) is an established alternative to surgery in patients with symptomatic severe aortic stenosis and has expanded its indications to even low-surgical-risk patients. Conduction abnormalities (CA) and permanent pacemaker (PPM) implantations remain a relatively common finding post TAVI due to the close proximity of the conduction system to the aortic root. New onset left bundle branch block (LBBB) and high-grade atrioventricular block are the most commonly reported CA post TAVI. The overall rate of PPM implantation post TAVI varies and is related to pre- and intra-procedural factors. Therefore, when screening patients for TAVI, Heart Teams should take under consideration the various anatomical, pathophysiological and procedural conditions that predispose to CA and PPM requirement after the procedure. This is particularly important as TAVI is being offered to younger patients with longer life-expectancy. Herein, we highlight the incidence, predictors, impact and management of CA in patients undergoing TAVI.

## 1. Introduction

Aortic stenosis (AS) is the third most frequent cardiovascular disorder in ages over 60 years, following atherosclerotic disease and hypertension [1]. Although rheumatic valvulopathy is uncommon in developed countries, the global burden of AS is increasing due to aging and population growth. Indeed, various epidemiological data describe an exponential increase in AS prevalence with advancing age, ranging from 0.2% in the 50–59 year group, 1.3% in the 60–69 year group, 3.9% in of the 70–79 year group and up to 9.8% in those aged 80–89 years [2].

Transcatheter aortic valve implantation (TAVI) is the gold standard treatment approach to symptomatic severe AS in patients deemed to be at high surgical risk and indications have expanded to intermediate and low-risk patients [3]. However, despite recent advancements in TAVI implantation techniques and device technology, the occurrence of new impulse conduction abnormalities (CA), including high-degree atrioventricular block (HAVB) requiring permanent pacemaker (PPM) implantation, represents one of the main procedural complications [4,5]. This is particularly relevant as TAVI is now being offered to younger patients.

## 2. Incidence of New Onset CA and PPM Implantation Post TAVI

The occurrence of new-onset CA post TAVI varies widely, ranging from 5% to 65% depending on the implanted device and applied techniques [6]. Recently published data from randomized trials and registries revealed that the risk of CA requiring PPM implantation lies between 6.7% and 39.2%, with a mean incidence of 19% [7]. The highest incidence of HAVB was observed in patients with preexisting right bundle branch block (RBBB) (13.2%) and in those with new-onset first degree atrioventricular (AV) block (13.9%). However, almost half of these conduction disturbances (CDs) may resolve over time, when inflammation and edema due to tissue manipulation have subsided, regardless of the prosthesis implanted. The prevalence of PPM implantation post TAVI ranged between 2% and 36% in a large meta-analysis comprising data from 17,139 patients. Of note, significant differences were observed depending on which device was utilized; balloon expandable valves ranged from 4 to 24%, whereas self-expanding ones were from 16.3 to 37.7% for the CoreValve™ (Medtronic, Minneapolis, MN, USA) and 14.7–26.7% for the Evolut™ (Medtronic, Minneapolis, MN, USA) series [8].

The above reported wide-ranged rates of events may also, in part, reflect the heterogeneity of CA endpoints and the lack of standardized criteria for PPM implantation [6,9,10]. According to the VARC-3 definitions, CA are classified based on the time of onset, as early (within 24 h of surgery) or late (>24 h of surgery) [11]. Regarding the timing of CA, the vast majority (>90%) occurred within the first week after the procedure, with the median day of PPM implantation being the third day. Based on recently published prospective multicenter data, 76% of the PPM-requiring events were observed within 4–6 days post TAVI, with similar timing patterns across the prespecified groups. On the other hand, in patients with pre-existing RBBB, the vast majority of high-degree CA occurred within 3 days post TAVI, and most of them had not recovered at the 1-month follow-up [12]. Moreover, compared to surgical aortic valve replacement (SAVR), the occurrence of CA leading to PPM implantation seems to happen a couple of days later in TAVI cases. Delayed HAVB may occur in up to 7% of the cases, being infrequent in patients with normal electrocardiogram (ECG).

## 3. Risk Factors for CA Post TAVI

### 3.1. Anatomic Factors

The AV node is located within the Koch’s triangle, which lies in the superficial paraseptal endocardium of the right atrium, and the emanating fibers subsequently form the bundle of His within the infra-anterior portion of the membranous septum (MS), which is in turn divided in the right and left bundle branches. The left bundle runs proximal to the base of the commissure between the non and right coronary cusp. Interindividual variations in the location of the bundle branches with respect to the membranous and muscular septum may explain the different risks of CA among patients [13].

Additionally, in patients with shorter MS length and thus a short distance between the His bundle and the aortic annulus, the risk of HAVB and PPM implantation was higher compared to those with longer length [14]. According to the MIDAS (Minimizing Depth According to the MS) technique, patients are classified into low (length > 5 mm), medium (length 2– 5 mm), or high risk (length <2 mm). Observational data have shown that patients with bicuspid aortic valves (BAV) have significantly shorter MS length and thus a higher risk of developing new LBBB, or requiring PPM post TAVI [15]. Therefore, MS is considered to be a surrogate marker for the location of the AV bundle; however, given the aforementioned anatomic variability, it may not be a reliable approach in a significant proportion of patients.

Another important predictor of PPM implantation after TAVI is an asymmetrically calcified aortic valve. The presence of a high calcium load on the left and non-coronary cusps is a potent factor for PPM implantation post TAVI [16]. This is likely attributable to an unequal distribution of radial forces on the aortic annulus and its adjacent structures, shifting the bio-prosthesis away from calcification, towards the RCC (where the bundle of His is located) [17]. The calcium distribution pattern is, thus, another important feature to be considered pre-TAVI.

### 3.2. Baseline Electrocardiography (ECG)

ECG is a readily available and effective tool to assess and predict post-procedural CA and the risk of PPM implantation, with baseline CA being the most powerful predictor. A large meta-analysis including 239 studies with a total of 981,168 patients confirmed that the most relevant predictors for PPM implantation were pre-existing RBBB (RR, 3.12; *p* < 0.001), bi-fascicular block (RR, 2.40; *p* = 0.002) and isolated 1st-AVB (RR 1.44; *p* < 0.001) [18]. A recent retrospective observational single-center study with 720 consecutive patients who underwent TAVI showed that R-wave amplitude in lead V1 during baseline ECG in patients with normal QRS duration may predict the occurrence of HAVB following new LBBB post TAVI [19]. Interestingly, post-procedural bradyarrhythmic events are not necessarily TAVI-related and may be pre-existent. Twenty-four-hour ECG monitoring on the preprocedural day can detect new arrhythmias in 16.1% of patients and, among those who ultimately required post-procedural PPM, 31.4% had newly diagnosed HAVB or severe bradycardia pre-TAVI [20]. PPM implantation at late post-TAVI is uncommon and is associated with clinical symptoms in 50% of the cases [21].

Various scoring models for the prediction of PPM implantation post TAVI based on ECG criteria have been verified. In 2019, the Emory Risk Score was developed for the prediction of PPM implantation post TAVI [22]. Variables included were a history of syncope, RBBB, QRS interval ≥ 140 ms, valve oversizing ≥16% with an area under the receiver-operating characteristic (ROC) curve of 0.778 (*p* < 0.001) and an odds ratio of 2.2 per point increase (*p* < 0.001). More recently, another scoring system was introduced: transfemoral approach, LBBB without bradycardia, sinus bradycardia without LBBB, RBBB, LBBB with sinus bradycardia and 2nd -AVB taken into calculation with the area under the curve of 0.6743 (95% CI: 0.618 to 0.729). The risk for PPM was stratified as follows: 7% risk of PPM with a score ≤ 3, 19% with a score 4 to 6 and 38% with a score ≥ 7 [23].

### 3.3. Demographic Characteristics

Although divergent data have been reported, data from meta-analyses support the concept of a sex-associated risk of PPM implantation post TAVI, with males at higher risk. Although women have a higher risk of in-hospital mortality and vascular complications, men are more likely to require PPM implantation [13,24]. However, recent data from a retrospective study by the Netherlands Heart Registration contradicted the above, suggesting a protective role for male sex against PPM implantation, possibly due to larger aortic annuli and thus the reduced occurrence of oversizing [25].

The role of age as a predictor was studied in large national registries, such as in recent reports from France and Switzerland, which reported that older age was associated with higher PPM implantation risk [14,26]. Likewise, a sub-analysis of the PRAGMATIC registry found age to be predictive of PPM implantation (OR 1.08, 95% CI: 1.04–1.12, *p* < 0.0001) [27].

### 3.4. Transcatheter Aortic device

There are significant differences between the transcatheter device and the risk of PPM (Table 1). A recent network meta-analysis analyzing 46,000 patients with post-TAVI PPM implantation revealed that (a) the implantation of balloon-expandable valves was associated with 39% and 62% lower PPM implantation rates compared to self-expanding and mechanically expanding ones, respectively; (b) the implantation of SEVs was associated with a 38% lower PPM implantation rate compared to MEVs; and (c) the ACURATE neo™ valve (Boston Scientific, MA, USA) was associated with the lowest post-TAVI PPM implantation rates [28]. A large meta-analysis demonstrated only 7.7% new PPM for the ACURATE neo due to its low radial force and predictable supra-annular deployment [29]. In the Evolut low-risk trial, 17.4% of patients received PPM, and the percentage was 6.6% in the PARTNER 3 trial [3]. Other large comparative meta-analyses confirm these notable differences among valve types.

Other predisposing factors for PPM implantation post TAVI are valve oversizing and high prosthesis/LVOT diameter ratio, leading to overstretching of the latter [5]. Pre-dilatation has historically been considered a mandatory step during TAVI to facilitate device crossing, deployment and optimal expansion. However, pre-dilation increases the annular trauma, and its role is still debatable. Randomized trials with the Evolut and Sapien 3 valves demonstrated non-inferiority of the direct TAVI with the pitfall of possibly more post-dilatations for the former device [30]. Balloon post-dilatation, though, may be potentially associated with PPM implantation and should be carefully considered [30].

### 3.5. Implantation Depth

Valve implantation depth is a well-known procedural risk factor for new-onset CA. Jilaihawi et al. proposed an individualized, anatomically guided tool for minimizing implantation depth according to a CT-measured MS (MIDAS strategy) as an important strategy to reduce CDs during TAVI [24]. The researchers found that high valve implantation at a depth shallower than the MS significantly reduced PPM implantation rates (from 9.7% to 3%) and new onset LBBB (from 25.8% to 9%). Less CA with low-depth deployment was also confirmed recently by Ochiai et al. with the drawback of higher coronary ostia obstruction rates [31]. The cusp-overlap projection is another technique facilitating a controlled implantation depth in case of SEVs. Cusp-overlap projection has the advantage of LVOT “elongation”, gaining a higher implantation depth (<3 mm), thus increasing the device positioning accuracy [24]. A recent propensity score analysis revealed an almost 50% reduction in PPM implantation rates with the cusp overlap technique versus the standard 3-cusp coplanar projection (*p* = 0.03) [32]. Data of the Optimize PRO study revealed improved safety and lower PPM implantation rates (9.8%, at 30 days) when the cusp overlap technique was used [33]. In addition, the deployment of balloon-expandable devices using the cusp overlap technique led to a more than 50% reduction in new LBBB occurrence and PPM implantation rates compared to standard projection (*p* < 0.001). On the other hand, balloon-expandable valves, characterized by short frame height, are usually deployed perpendicularly to the aortic annulus when the standard 3-cusp coplanar projection is used, thus minimizing the interference of the device with the anatomy of the conduction system.

### 3.6. Medication

Various medications may increase the risk of CDs and/or the need for PPM implantation following TAVI. These include antiarrhythmics, antihypertensives, psychoactives/neuroleptics and anticancer drugs (5-fluorouracil, cyclophosphamide, anthracycline, etc.) [34]. Several publications address the topic of beta blocker (BB) discontinuation in patients undergoing TAVI, yet it has not been adequately clarified whether the periprocedural continuation of BBs increases NOCDs and PPM implantation following TAVI, although there is indeed a tendency to withdraw them or to reduce the dose for PPM implantation prevention. In fact, the OCEAN registry showed similar rates of PPM implantation and better cardiovascular outcomes for the BB arm [35]. Moreover, prospective data revealed that the rate of periprocedural HAVBs and thus PPM implantation was lower among patients who continued BB versus those who did not (20% vs. 13%; *p* = 0.02). Interestingly, a multivariate analysis of the above-mentioned study revealed that the risk of periprocedural arrhythmic events is double in those who discontinue BB [36]. The BETA TAVI (NCT05721170), a prospective, multicenter RCT, will provide more solid data regarding the role of BBs in TAVI population.

## 4. Impact of New LBBB, New CA and PPM Implantation

As TAVI is being offered to younger and lower-risk patients, the impact of PPM implantation and dependency on overall mortality is becoming a matter of debate. Recently, data from Sweden revealed that there was no significant difference in long-term survival up to 10 years between patients with and without new PPM implantation (HR 1.03,95% CI 0.88–1.22; *p* = 0.692) [37]. Correspondingly, in a multicenter study including 1.020 patients undergoing TAVI, new LBBB was not associated with increased mortality after a median follow-up of 3 years [38]. Studies and meta-analyses revealed that the 12-month all-cause mortality rate was similar among patients without PPM (18.0%), patients with PPM before TAVI (22.9%) and patients with PPM after TAVI (19.4%) [39]. In contrast, a recent Danish single-center study showed higher 5-year mortality in patients with new LBBB (48.4%; HR 1.79, 95% CI 1.24–2.59) and new PPM implantation (46.7%; HR 1.58, 95% CI 1.01–2.46) versus those without new CA [40]. A meta-analysis of 12 TAVI studies revealed that both new onset LBBB and PPM implantation may be associated with an increased risk of all-cause death and heart failure hospitalization at 1-year follow-up [10]. Similarly, in a propensity score analysis of a large SAVR study with a mean age of 70 years, overall mortality was significantly higher in patients with new PPM implantation versus those without (HR 1.14, 95% CI 1.01–1.29; *p* = 0.03) [41]. In another study, after multivariable adjustment, the impact on all-cause mortality was statistically significant for new LBBB, whereas the association did not reach the conventional level of statistical significance for new PPM implantation [42]. Also, recent data from the STS/TVT Registry encompassing 9.785 TAVI recipients revealed a higher risk in one-year all-cause mortality among patients who had a PPM after TAVI (adjusted hazard ratio 1.31; 95% CI: 1.09–1.58; *p* = 0.003) [43].

As shown above, data on the impact of CA and PPM implantation on patient outcomes post TAVI remain conflicting. Both right ventricular apical pacing and LBBB may cause LV dyssynchrony, resulting in adverse LV remodeling. This association between new LBBB/new PPM and adverse LV remodeling has also been suggested in TAVI patients [44,45]. Biventricular, His-bundle or physiological pacing may decrease such dyssynchronous ventricular pacing by attenuating the PPM-induced adverse effect post TAVI [40,46]. LBBB-area pacing is an emerging technique that has demonstrated promising results comparable to conventional epicardial biventricular pacing, constituting a possible alternative [47].

## 5. Current and Future Strategies to Address CA and the Need for PPM Implantation

**Figure 1:**
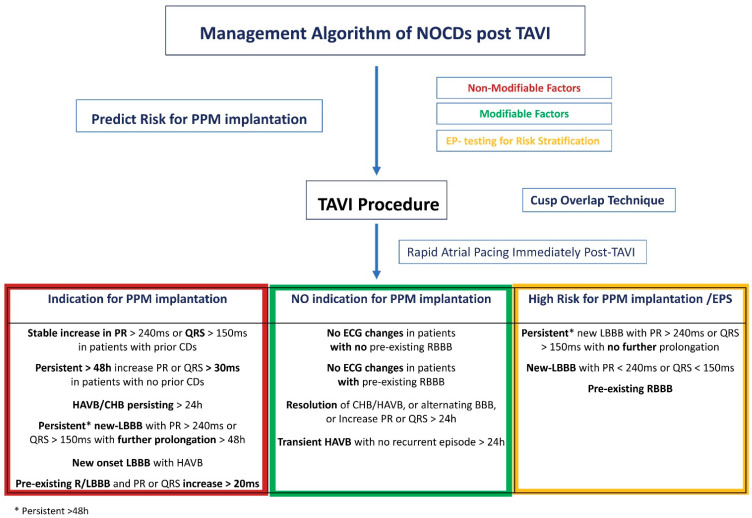
Proposed management algorithm of new-onset conduction abnormalities and need for permanent pacemaker implantation after transcatheter aortic valve implantation.

Currently, there is no defined universal ideal timing for PPM implantation post TAVI. This could be challenging since some periprocedural CA are transient or present with delay. Systematic 2-week AECG monitoring following TAVI revealed that, although delayed HAVB/CHB post TAVI is rare in patients without ECG changes, baseline RBBB and new-onset CAs suggest an increased risk, especially in deeper device implantations and when predilating had occurred [12]. Based on the available clinical trials, expert consensus documents with structured diagnostic and management approaches have been published [10,34,48,49]. Herein, we propose such a management algorithm for new CDs post TAVI (Figure 1).

Strategies for stratifying patients who may benefit from extended inpatient rhythm monitoring, such as a rapid atrial pacing (RAP) test post TAVI, as well as from performing an electrophysiologic study (EPS), have been described. Indeed, the absence of Wenckebach heart block upon RAP from 70 to 120 bpm was associated with a nearly 99% negative predictive value for post-TAVI PPM requirement [50]. On the other hand, this approach did not show any added predictive value in another observational study, leaving questions about the proper application of these potentially promising tests, revealing the need for further research [51]. This individualized approach allows for a degree of safety for early post-TAVI discharge. EPS can be used to evaluate patients with uncertain indications of PPM post TAVI. It is known that TAVI is a cause of post-procedural prolongation of the His-Ventricular (H-V) intervals; however, there are no established cutoff H-V interval prolongation values that could predict the occurrence of a third-degree AV block. Recently, a cutoff H-V interval > 100 ms with or without procainamide challenge for PPM implantation has been proposed [52]. This EPS-guided strategy led to a 70% reduction in PPM rates in the cohort of patients with equivocal indications without increasing the length of hospital stay or mortality. Knecht et al. have shown that EPS can be used to identify patients with new LBBB post TAVI who will not develop third degree AV block if they had an H-V interval < 55 ms with a negative predictive value of 90% [53]. Therefore, large randomized trials are needed to further evaluate the utility of EPS in identifying the proper HV interval threshold.

Hybrid imaging approaches, allowing the fusion of transesophageal echocardiogram and/or multislice computed tomography with fluoroscopy, can improve 3D imaging [54]. However, these approaches do not visualize the conduction system, which is characterized by great anatomical variation. Thus, imaging approaches demonstrating electrical pathways and 3D soft tissue anatomy, in combination with new valve technologies, may be beneficial.

## 6. Conclusions

New-onset CAs remain one of the main limitations of TAVI, despite the progress in implantation techniques and device technology. Based on recent data, several predictors of CAs and algorithms of management embracing an individualized approach have been proposed. However, further research is warranted in order to minimize this “Achilles’ heel” of TAVI. Until then, detailed screening and management approaches are needed in order to eliminate the risk, as TAVI is being offered to younger patients with longer life-expectancy.

## 7. Key Points

- The occurrence of new-onset LBBB and conduction abnormalities, including high-grade atrioventricular block requiring permanent pacemaker implantation, remain the most common complications following TAVI.
- The Heart Team’s choice between SAVR and TAVI should also weigh the risk of a temporary pacemaker, especially in young patients.
- The existence of RBBB is the strongest predictor of permanent pacemaker implantation post TAVI.
- The risk of new-onset conduction abnormalities requiring pacemaker implantation is related to the pre-procedural, intra-procedural and device characteristics.
- Post-procedural care requires a stepwise approach regarding temporary pacemaker presence duration, arrhythmic monitoring and permanent pacemaker implantation decisions.

## Figures and Tables

**Table 1:** The incidence of permanent pacemaker implantation after transcatheter aortic valve implantation in major randomized clinical trials.

Clinical Trial	Studied Valve Type	STS Score, (%)	Age, (Years)	30-Day Pacemaker Rate, (%)
**US CoreValve**	CoreValve	7.3 ± 3.0	83.2 ± 7.1	19.8
**SURTAVI**	CoreValve, Evolut R	4.4 ± 1.5	79.9 ± 6.2	25.9
**Evolut Low Risk**	CoreValve, Evolut R/PRO	1.9 ± 0.7	74.0 ± 5.9	17.4
**Notion**	CoreValve	2.9 ± 1.6	79.2 ± 4.9	34.1
**Scope I**	ACURATE neo	3.7 (2.5–4.9)	82.6 ± 4.3	10
**Scope II**	ACURATE neo	4.6 (3.0)	83.4 (4.2)	11
**Partner**	Sapien	11.8 ± 3.3	83.6 ± 6.8	3.8
**Partner 2**	Sapien XT	5.8 ± 2.1	81.5 ± 6.7	8.5
**Partner 3**	Sapien 3	1.9 ± 0.7	73.3 ± 5.8	6.6
**Portico IDE**	Portico	6.4 (3.4)	83.0 (7.6)	27.7

## Data Availability

Data is contained within the article.

## Abbreviations

**AV**: Atrioventricular; **AS**: Aortic Stenosis; **CA**: Conduction Abnormalities; **ECG**: Εlectrocardiogram; **HAVB**: High-Degree Atrioventricular Block; **HV**: His-Ventricular; **LBBB**: Left Bundle Branch Block; **MS**: Membranous Septum; **PPM**: Permanent Pacemaker; **RBBB**: Right Bundle Branch Block; **SAVR**: Surgical Aortic Valve Replacement; **TAVI**: Transcatheter Aortic Valve Implantation.
